# Perfluoro Alkyl Hypofluorites and Peroxides Revisited

**DOI:** 10.1002/chem.201903620

**Published:** 2019-10-23

**Authors:** Jan H. Nissen, Thomas Drews, Benjamin Schröder, Helmut Beckers, Simon Steinhauer, Sebastian Riedel

**Affiliations:** ^1^ Fachbereich für Biologie, Chemie, Pharmazie Institut für Chemie und Biochemie—Anorganische Chemie Freie Universität Berlin Fabeckstraße 34/36 14195 Berlin Germany

**Keywords:** gas-phase fluorine chemistry, hypofluorites, perfluoro bisalkyl peroxides, vibrational spectroscopy

## Abstract

A more convenient synthesis of the perfluoro alkyl hypofluorite (F_3_C)_3_COF as well as the hitherto unknown (C_2_F_5_)(F_3_C)_2_COF compound is reported. Both hypofluorites can be prepared by use of the corresponding tertiary alcohols R^F^OH and elemental fluorine in the presence of CsF. An appropriate access to these highly reactive hypofluorites is crucial. The hypofluorites are then transferred into their corresponding perfluoro bisalkyl peroxides R^F^OOR^F^ [R^F^=(F_3_C)_3_C, (C_2_F_5_)(F_3_C)_2_C] by treatment with partially fluorinated silver wool. NMR, gas‐phase infrared, and solid‐state Raman spectra of the perfluoro bisalkyl peroxides are presented and their chemical properties are discussed.

## Graphical abstract

**Once upon a time… Hypofluorites**: The convenient synthesis of alkyl hypofluorites R^F^OF [R^F^=(F_3_C)_3_C, (C_2_F_5_)(F_3_C)_2_C] is the origin story for perfluoro bisalkyl peroxides. Hypofluorites are prepared from the corresponding alcohols R^F^OH and elemental fluorine. The perfluoro bis‐*tert*‐butyl peroxide is highly inert towards oxidizers and even dissolves elemental fluorine without decomposition.

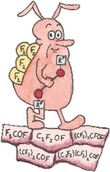

## Introduction

Cady and Kellogg described the synthesis of the first perfluoro alkyl hypofluorite, trifluoromethyl hypofluorite, F_3_COF, by the AgF_2_‐catalyzed direct fluorination of methanol in 1948.^1^ Since then, a variety of perfluoro^2^ as well as partially chlorinated (e.g., Cl_3_CCF_2_OF)^2^, ^3^ and nitrogen (e.g., NF_2_CF_2_CF_2_OF)^4^ or sulfur (e.g., FSO_2_CF_2_CF_2_OF)^5^ containing and later even hydrogen (e.g., H_3_COF)^6^ substituted alkyl hypofluorites ROF (**1**) have been isolated and characterized. An alternative route to perfluoro alkyl hypofluorites is the fluorination of perfluoro ketones in the presence of metal fluorides, MF (M=K, Rb, Cs) [Eq. 1], as described in 1966 by Ruff et al.^7^ Moreover, the direct fluorination (with 10 % F_2_ in N_2_) of sodium trifluoroacetate, F_3_CCO_2_Na,^8^ or trifluoroacetic acid, F_3_CCO_2_H,^9^ leads to the formation of hypofluorite compounds in a temperature‐dependent ratio. These hypofluorites were used in the pioneering works by Hesse and co‐workers^10^ and Rozen^11^ as electrophilic fluorination agents or as etching gas^12^ for semiconductors. The relatively low dissociation energy of the O−F bond in F_3_COF (184.2 kJ mol^−1^)^13^ facilitates insertion of carbon monoxide^14^ into the O−F bond to produce fluoroformiates, F_3_COC(O)F, and the addition of F_3_COF to (per)fluorinated olefins, which yields (per)fluorinated bisalkyl ethers, F_3_COR.^15^ Further reactions of functionalized ethers such as F_3_COCFCl‐CF_2_Cl may then be reduced to the vinylether, F_3_COCF=CF_2_, which has become a valuable fluorinated monomer for the industrial synthesis of perfluoroxy alkanes (PFA).^16^

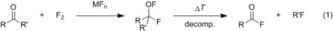



**Scheme 1:**
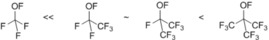
Increasing tendency of hydrolysis of selected perfluoro alkyl hypofluorites, R^F^OF.

Hypofluorites are highly hazardous compounds, which require sophisticated handling and safety precautions. High reactivity and oxidation power is often paired with an intrinsic instability, which may lead to a rapid exothermic decomposition upon contact with organic impurities or metal surfaces.^17^ Decomposition of R^F^OF to yield the corresponding carbonyl compounds [Eq. (1)] is highly exothermic with reaction enthalpies Δ*H*_R_ of up to −400 kJ mol^−1^ (see the Supporting Information, Table S2.1). The susceptibility to hydrolysis of perfluorinated hypofluorites, R^F^OF, producing alcohols, R^F^OH, increases with increasing numbers of fluorinated groups (Scheme 1). Trifluoromethyl hypofluorite, F_3_COF, is thermally rather stable up to temperatures above 450 °C and hydrolysis in aqueous solution is very slow.^1^ The chemistry of perfluoro alkyl hypofluorites has been recently reviewed.^17^

We found that perfluoroalkyl hypofluorites R^F^OF are excellent starting compounds for the synthesis of otherwise difficult to access perfluoro bisalkyl peroxides R^F^OOR^F^ (**2**),^18^ which are synthetically valuable sources of fluoroalkoxy radicals, R^F^O^.^.^19^ Until very recently,^18^ our knowledge about perfluoro bisalkyl peroxides was very scarce and limited to the two homologs bis(trifluoromethyl) peroxide, (F_3_CO)_2_ (**2 a**)^20^ and bis(nonafluoro‐*tert*‐butyl) peroxide, [(F_3_C)_3_CO]_2_ (**2 b**).^21^ The volatile and rather resistant peroxide **2 a** (bp. −37 °C) was reported in 1933 by Swarts^22^ and prepared in 1957 by Cady et al.^23^ from trifluormethyl hypofluorite and carbonyl difluoride, F_2_CO. One decade later, Anderson et al. detected peroxide **2 b** among other products obtained by the reaction of chlorine trifluoride and perfluoro *tert*‐butyl alcohol, (F_3_C)_3_COH (**3 b**) in a high‐pressure stainless‐steal reactor.^21^ Later, the low‐temperature photolysis (−40 °C) of perfluoro *tert*‐butyl hypofluorite, (F_3_C)_3_COF (**1 b**), in the presence of tetrafluorohydrazine, N_2_F_4_, was also described to yield peroxide **2 b**.^19^ The gas‐phase molecular structure of **2 a** was investigated by electron diffraction^20^ whereas the solid‐state structure of **2 b** has only very recently been published.^18^ The chemistry of these perfluoroalkyl peroxides has only been scarcely investigated in the past. Among them are reactions with carbon–carbon double bonds of fluorinated olefins^24^ and thiophenes.^25^ Furthermore, the closely related compound class of fluoroformyl peroxides, R^F^‐OO‐C(O)F,^26^ as well as perfluorodiacyl peroxides, R^F^C(O)‐OO‐C(O)R^F^, have been investigated and represent important intermediates in the industrial synthesis of fluorous polyether chains.^27^

Here, we report a modified synthesis of tertiary perfluoroalkyl hypofluorites R^F^OF [R^F^=(F_3_C)_3_C **1 b**, R^F^=(C_2_F_5_)(F_3_C)_2_C **1 c**] from the corresponding alcohols R^F^OH [R^F^=(F_3_C)_3_C **3 b**, R^F^=(C_2_F_5_)(F_3_C)_2_C **3 c**] with elemental fluorine in the presence of excess CsF. These hypofluorites **1** were then treated with fluorinated silver wool to form the perfluoro bisalkyl peroxides R^F^OOR^F^ [R^F^=(F_3_C)_3_C **2 b**, R^F^=(C_2_F_5_)(F_3_C)_2_C **2 c**].

## Results and Discussion

Perfluoroalkyl hypofluorites, R^F^OF (**1**), were obtained according to Equation (1) by treatment of the corresponding carbonyl compound with elemental fluorine in a stainless‐steel reactor in the presence of any alkali metal fluoride, MF. The mixture was initially cooled to −196 °C, and then slowly heated to −78 °C.^17^, ^28^, ^29^ Because these mixtures are highly hazardous and may explode spontaneously,^28^ we first improved this synthesis to avoid any possible local heat formation and pressure increases during the reaction.

The number of perfluoroalkyl hypofluorites **1** obtainable by this procedure can be further increased by using perfluoro alkylalcohols **3** instead of the perfluoro carbonyl compounds according to Equation 2. First, the perfluoro alkylalcohols **3 b**,**c** were added to an excess of cesium fluoride in a stainless‐steel reactor and the mixture was thoroughly shaken at room temperature to dissipate the reaction heat of the subsequent fluorination reaction. It is assumed that the alcohol reacts with CsF to give the corresponding cesium alcoholate and CsHF_2_.^29^ The reactor was cooled to −78 °C again and elemental fluorine was added in small portions via a stainless‐steel line until no fluorine was consumed anymore and the pressure remained constant. Excess fluorine was then removed from the reaction mixture at −196 °C and the hypofluorites **1** thus formed were distilled out of the reactor and purified by trap‐to‐trap distillation. For the hypofluorites (F_3_C)_3_COF (**1 b**) and (C_2_F_5_)(F_3_C)_2_COF (**1 c**) the conversion is quantitative.
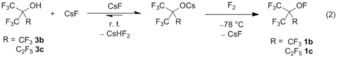



This fluorination procedure was finally also used for the synthesis of the known hypofluorites F_3_COF (**1 a**), CF_3_CF_2_OF (**1 d**), and (F_3_C)_2_CFOF (**1 e**) starting from the perfluoro carbonyl compounds F_2_CO, F_3_CC(O)F, and (F_3_C)_2_CO, respectively, according to Equation (1). Their IR and NMR spectra match those previously reported.^2^ Quantum‐chemical calculations indicate that apart from **1 a**, all these perfluoro alkyl hypofluorites are thermodynamically unstable by up to −400 kJ mol^−1^ in terms of elimination of CF_4_ and formation of the corresponding carbonyl compounds (see the Supporting Information, Table S2.1). As expected, elimination of elemental fluorine is endothermic by 120–150 kJ mol^−1^. Nevertheless, these perfluoro alkyl hypofluorites are kinetically stable in the gas‐phase up to 110 °C.^2^ Therefore, we were able to provide atmospheric pressure chemical ionization (APCI) mass spectra of *tert*‐butyl hypofluorite **1 b** (see the Supporting Information, Figure S1.2). The predominant species in the negative mode is the alkoxide ion [(F_3_C)_3_CO]^−^ (*m*/*z =* 235), whereas the molecular ion peak at *m*/*z =* 254 is not observed. Interestingly, the peak at *m*/*z =* 285 can be assigned to an adduct or insertion of a CF_2_ fragment to the alkoxide ion [(F_3_C)_3_CO+CF_2_]^−^, which was previously observed for a perfluorinated ether bearing the perfluoro‐*tert*‐butyl group.^30^ Only perfluoro *tert*‐pentyl hypofluorite (**1 c**), which is stable in solution for several minutes, is found to decompose readily at room temperature within seconds to yield selectively (F_3_C)_2_CO and C_2_F_6_, as proved by their gas‐phase IR spectra [Eq. 3].^31^, ^32^

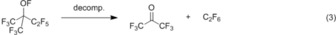



**Figure 1:**
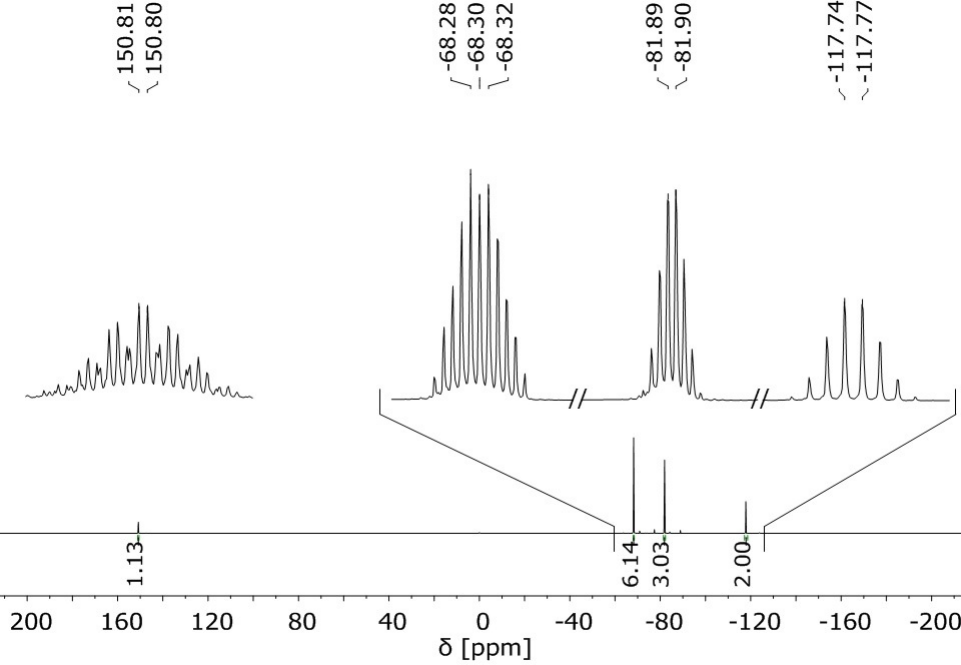
^19^F NMR spectrum of (C_2_F_5_)(F_3_C)_2_COF (**3 c**; 376.88 MHz, external [D_6_]acetone, −60 °C).

Probably as a result of the lack of stability, hypofluorite **1 c** has not yet been characterized.^10^, ^33^ We therefore present low‐temperature NMR as well as gas‐phase IR spectra of this poorly known compound. These spectra clearly confirm the presence of **1 c**. The fully coupled ^13^C NMR spectrum (see the Supporting Information, Figure S1.3) shows two quartets at *δ*=118.5 ppm (C‐*C*F_3_) and *δ*=116.8 ppm (F_3_ *C*‐CF_2_), where the latter one partly overlaps with the *C*F_2_ triplet at *δ*=109.8 ppm. The chemical shift of the quaternary carbon atom appears at *δ*=87.7 ppm. The first‐order ^19^F NMR spectrum of hypofluorite **1 c** (Figure 1) shows a doublet of septets for the C*F*_2_ nuclei at *δ*=−117.8 ppm, indicating a ^4^ *J*(F,F) coupling to O*F* and to the two C*F*_3_ nuclei of 11.6 Hz. The *F*_3_CCF_2_ signal at *δ*=−81.9 ppm also shows a doublet of septets with similar coupling constants of ^5^ *J*(F,F)=5.9 Hz to both the O*F* and the C‐C*F*_3_ nuclei. The signal for the two C*F*_3_ groups occurs as a doublet of triplet of quartets (d t q) at *δ*=−68.3 ppm with a large ^4^ *J* coupling constant of 17.7 Hz to the O*F* nucleus, which resonates at *δ*=150.8 ppm (t sept q). The ^3^ *J* coupling constant between the fluorine atoms of the pentafluoroethyl group is smaller than 0.5 Hz.

**Figure 2:**
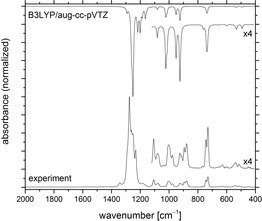
Gas‐phase IR spectrum of (C_2_F_5_)(F_3_C)_2_COF (**3 c**; bottom) and the calculated spectrum for the most stable *t*‐1 conformer (see Figure 3) at the B3LYP/aug‐cc‐pVTZ level of theory (top).

**Figure 3:**
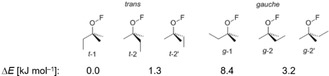
Relative energies of *trans* and *gauche* conformers of (C_2_F_5_)(F_3_C)_2_COF (**1 c**) obtained at the B3LYP/aug‐cc‐pVTZ level of theory (fluorine atoms bound to carbon are not shown).

The IR spectrum of hypofluorite **1 c** in the gas phase is compared to a computed IR spectrum at the DFT‐B3LYP/aug‐cc‐pVTZ level of theory in Figure 2. The experimental bands in the mid‐IR range from 1107 to 878 cm^−1^ are split into two components, probably owing to the presence of at least two rotational conformers in the gas phase. Indeed, the DFT calculations revealed slightly different IR spectra for the different *trans* and *gauche* rotational conformers of **1 c** (Figure 3, Table 1, and the Supporting Information) and a global minimum for the *t*‐1 structure. This result agrees well with the experimental IR spectrum, which shows strong absorption for the C−F stretching modes in the region from 1291 to 1232 cm^−1^ and the characteristic deformation bands of the CF_3_ groups at 766, 743, and 730 cm^−1^. Furthermore, the bands at 1107 and 1078 cm^−1^ are tentatively assigned to C−O stretching modes of different rotamers of **1 c**. According to the calculations, the O−F stretching mode has a relatively low intensity and its position varies by up to 43 cm^−1^ depending on the conformer of **1 c** (see Table 1 and the Supporting Information). It can tentatively be assigned in the experimental gas‐phase IR spectrum to weak absorptions at 925 and 913 cm^−1^, but we cannot exclude that this band is superimposed by the asymmetric stretching mode of the C‐(CF_3_)_2_ fragment located at 1003 cm^−1^. The bands at 895 and 878 cm^−1^ represent C−C stretching modes of the pentafluoroethyl group of **1 c**. The calculated position of this band varies for the different conformers by 36 cm^−1^. The weak band at 613 cm^−1^ can be associated with the CC_3_ deformation, whereas weaker deformation modes of the CF_3_ group are found at 539 and 513 cm^−1^. The very weak absorption at 484 cm^−1^ fits well to a computed deformation mode of the C_2_F_5_ group.

**Table 1:** Gas‐phase vibrational frequencies $\tilde{v}$ [cm^−1^] and relative IR band intensities^[a]^ compared with computed values for different rotational conformers of (C_2_F_5_)(F_3_C)_2_COF (**1 c**) at the B3LYP/aug‐cc‐pVTZ level of theory.^[b]^

Experiment	DFT		Assignment
	*t*‐1	*g‐*1	
1342 (m)	1290 (75)	1308 (22)	*ν*(F_2_C‐CF_3_)
1291 (s, sh)	1273 (272)	1260 (421)	*ν*(CF_3_)
1276 (vs)	1248 (606)	1246 (541)	*ν*(CF_3_)
1262 (vs)	1239 (403)	1237 (338)	*ν*(CF_3_)
1255 (vs)	1233 (58)	1221 (157)	*ν*(CF_3_)
1251 (vs)	1218 (110)	1216 (240)	*ν*(CF_3_)
1232 (s)	1197 (297)	1201 (256)	*ν*(CF_3_)
1181 (m)	1161 (25)	1155 (5)	*ν*(CF_3_)
1161 (w, sh)	1154 (63)	1153 (11)	*ν*(CF_2_)
1107 (m), 1078 (w)	1064 (121)	1113 (14)	*ν*(CO)
1038 (vw)	1055 (12)	1047 (49)	*ν*(C‐CF_2_)
1003 (m), 977 (w)	972 (100)	963 (93)	*ν*_as_(C‐(CF_3_)_2_)
925 (w), 913 (w)	995 (25)	967 (34)	*ν*(OF)
895 (m), 878 (m)	888 (117)	898 (150)	*ν*(F_2_C‐CF_3_)
766 (vw)	766 (5)	765 (1)	*δ*(CF_3_)
743 (m)	739 (59)	740 (59)	*δ*(CF_3_)
730 (m)	725 (39)	725 (40)	*δ*(C(CF_3_)_2_)
613 (vw)	619 (6)	621 (12)	*δ*(CC_3_)
558 (sh)	537 (4)	537 (3)	*δ*(CF_3_)
539 (vw)	525 (9)	527 (10)	*δ*(CF_3_)
513 (vw)	501 (7)	499 (8)	*δ*(CF_3_)
484 (vw)	444 (2)	445 (3)	*δ*(C_2_F_5_)

[a] Relative intensities in parentheses: vw=very weak, w=weak, m=medium, s=strong, vs=very strong, sh=shoulder. [b] For the different *trans* and *gauche* rotational conformers see Figure 3 and Figure S2 in the Supporting Information.

The hypofluorites **1 b** and **1 c** are converted in rather good yield (up to >70 %) into the corresponding symmetric perfluoro bisalkyl peroxides **2 b** and **2 c**, respectively, by using partially fluorinated silver wool [see the Experimental Section and Eq. 4]. This reaction does not proceed at low temperatures of −78 °C, whereas at 0 °C mainly decomposition products of the hypofluorites are formed [**1 b**: CF_4_, (F_3_C)_2_CO; **1 c**: C_2_F_6_, (F_3_C)_2_CO].^31^, ^32^, ^34^ When the reaction vessel is held at temperatures of −50 to −45 °C for 48 to 72 h, peroxide **2 b** is obtained from **1 b** and can be separated by trap‐to‐trap distillation in a −78 °C trap from the more volatile side products CF_4_ and (F_3_C)_2_CO in an overall yield of >70 %. Pure peroxide **2 b** is rather stable at ambient temperature and decomposes at temperatures above 100 °C to yield (F_3_C)_2_CO and C_2_F_6_ with an activation energy of 148.7±4.4 kJ mol^−1^.^35^ Similarly, hypofluorite **1 c** reacts to give the peroxide **2 c** and this was purified from the volatile side products C_2_F_6_ and (F_3_C)_2_CO by trap‐to‐trap distillation, where it remains in a −78 °C trap in a yield of up to 66 %.
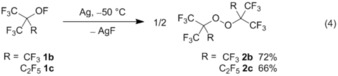



However, attempts to convert the hypofluorites CF_3_CF_2_OF (**1 d**) and (F_3_C)_2_CFOF (**1 e**) with fluorinated silver wool under similar conditions to the corresponding perfluoro bisalkyl peroxides failed and led solely to decomposition products (**1 d**: CF_4_,^34^ F_2_CO,^36^ **1 e**: CF_4_,^34^ F_3_CC(O)F),^37^ which were identified by IR spectroscopy. The composition of the fluorinated silver wool used in the synthesis of the peroxides **2 b** and **2 c** [Eq. (4)] was prepared as described in the Experimental Section and was investigated by powder X‐ray diffraction analysis. It consists mainly of silver(I) fluoride, AgF, but also contains some silver subfluoride, Ag_2_F, silver(II) fluoride, AgF_2_, and elemental silver, Ag (see Figure S1.1 in the Supporting Information). However, attempts to reproduce the above described conversion of hypofluorites **1** to peroxides **2** by using either elemental Ag or commercial AgF instead of the fluorinated silver wool failed and only decomposition products of the hypofluorites were obtained.

The mechanism of this solid–gas reaction for the formation of peroxides from hypofluorites is still unknown and further studies are necessary to explore this reaction. Powder diffraction measurements of the fluorinated silver wool prior and after several batches (Figure S1.1 in the Supporting Information) indicates an increase in silver(I) fluoride at the expense of silver(0) or silver subfluorides within several reactions. From this result, it can be assumed that the active site of the partially fluorinated silver wool consists of an incompletely coordinated silver subfluoride or silver(0) species, which acts as a fluorine‐atom acceptor. We noticed that the fluorine‐atom acceptor ability of this species depletes, and thus, the yield of peroxide formation decreases after several successful batches, very likely owing to fluorination of the active silver species and formation of inactive silver(I) fluoride. Based on this assumption, the following reaction mechanism can thus be postulated for the solid–gas reaction. First, the active silver site may undergo an oxidative addition of R^F^OF to form an alkoxide silver(II) intermediate, which then decomposes into alkoxyl radicals, R^F^O**^.^**, and silver(I) fluoride [Eq. 5]. The free or loosely bound R^F^O**^.^** radicals may then combine to form the peroxides **2**.
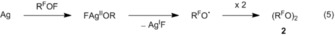



There are precedents for the formation of silver(II) alkoxides and their decomposition into alkoxyl radicals. Wechsberg and Cady previously described the reaction of AgF_2_ with F_2_CO and fluorine and assumed the formation of an Ag^II^(OR^F^)_2_ intermediate.^38^ Owing to the high oxidation potential of Ag^II^ (electron affinity: 21.45 eV),^39^ the proposed Ag^II^ alkoxide intermediates are prone to a ligand‐to‐metal electron‐transfer (LMCT) and even to the formation of alkoxyl radical intermediates such as Ag^I^(O**^.^**R^F^)(OR^F^).^39^ A similar radical mechanism has been proposed for the AgF_2_‐catalyzed low‐temperature reaction of F_2_ with SO_3_ to yield peroxy disulfuryl difluoride, (FSO_2_O)_2_.^40^

The ^13^C {^19^F} DEPTQ NMR spectrum^41^ of neat [(F_3_C)_3_CO]_2_ (**2 b**) shows two signals at *δ*=118.8 and 84.3 ppm associated with the *C*F_3_ and the quaternary carbon nuclei. The ^19^F NMR spectrum shows a singlet at *δ*=−69.6 ppm (lit.:^42^ −70.0 ppm) whereas the resonance in the ^17^O NMR spectrum occurred in the characteristic region of a peroxide compound^43^ at *δ*=246 ppm, see Figure S1.4 (in the Supporting Information). The oxygen atoms of (F_3_CO)_2_ (**2 a**) resonate at 262 ppm in the ^17^O NMR spectrum (Figure S1.5 in the Supporting Information). The base peak in the APCI mass spectrum of **2 b** (Figure S1.6 in the Supporting Information) at *m*/*z*=235 represents the [(F_3_C)_3_CO]^−^ fragment. Also, the [(F_3_C)_3_C]^−^ fragment can be assigned to the *m*/*z*=219 peak, whereas the molecular ion peak at *m*/*z*=470 is not present. The ^13^C {^19^F} DEPTQ NMR spectra of [(C_2_F_5_)(F_3_C)_2_CO]_2_ (**2 c**; Figure S1.7 in the Supporting Information) with optimized ^1^ *J* coupling constants of 290 Hz and 35 Hz, respectively, show the expected signals for the fluorine substituted carbon atoms at 118.5, 116.7, and 115.2 ppm and the resonance of the quaternary carbon atom at 85.6 ppm. They are slightly shifted to lower field compared with the corresponding hypofluorite **1 c**. Three signals observed in the ^19^F NMR spectrum (Figure S1.8 in the Supporting Information) are also shifted by Δ*δ*=2 ppm to lower field compared with the spectrum of the reactant **1 c**. This consistent low field shift of the NMR signals underlines the strong electron‐withdrawing effect of the perfluorinated *tert*‐pentyl group of peroxide **2 c**.

**Figure 4:**
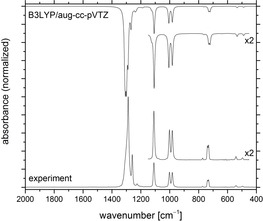
Gas‐phase IR spectrum of [(F_3_C)_3_CO]_2_ (**2 b**; bottom) in comparison to a computed spectrum at the B3LYP/aug‐cc‐pVTZ level (top).

The IR spectrum of [(F_3_C)_3_CO]_2_ (**2 b**) in the gas phase is shown in Figure 4 together with the computed spectrum at the DFT‐B3LYP/aug‐cc‐pVTZ level of theory. The strongest absorptions are associated with the CF_3_ stretching bands in the region around 1300 cm^−1^ (Table S1.1 in the Supporting Information). The sharp IR band at 1110 cm^−1^ is assigned to a C−O stretching mode whereas the second C−O stretch appears at 1129 cm^−1^ in the low‐temperature Raman spectrum (Figure S1.9 in the Supporting Information). The characteristic C−C_3_ stretching bands of the *tert*‐butyl group are found at 1002 and 982 cm^−1^ in the IR spectrum. This agrees well with their computed band positions at the DFT‐B3LYP/aug‐cc‐pVTZ level of theory at 994 and 970 cm^−1^, respectively. The corresponding Raman band shows a strong absorption at 1027 cm^−1^. The calculated O−O stretching mode at 902 cm^−1^ can clearly be assigned to a Raman band at 865 cm^−1^. Very strong Raman bands are also found for the symmetric CF_3_ deformation modes at 783 and 749 cm^−1^. Their counterparts in the gas‐phase IR spectrum of peroxide **2 b** appear at 739 and 731 cm^−1^. The asymmetric CF_3_ deformation modes are located at 541 and 496 cm^−1^ in the IR spectrum and at 569, 541, and 523 cm^−1^ in the Raman spectrum. A characteristic deformation of the C−O−O−C peroxide moiety is assigned to a Raman band at 356 cm^−1^, close to the CF_3_ rocking modes in the region from 339 to 296 cm^−1^. Two strong Raman bands at 241 and 123 cm^−1^ represent the CC_3_ deformation modes of [(F_3_C)_3_CO]_2_ (**2 b**).

**Figure 5:**
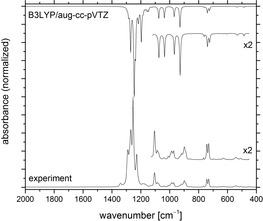
Gas‐phase IR spectrum of [(C_2_F_5_)(F_3_C)_2_CO]_2_ (**2 c**; bottom) in comparison to a computed spectrum at the B3LYP/aug‐cc‐pVTZ level (top).

Figure 5 shows the IR spectrum of [(C_2_F_5_)(F_3_C)_2_CO]_2_ (**2 c**) together with the computed spectrum at the DFT‐B3LYP/aug‐cc‐pVTZ level of theory. As expected, it is very similar to the spectrum of **2 b** and to that of its precursor **1 c**. The IR spectrum shows, in addition to the strong CF_3_ stretching modes in the region from 1277 to 1229 cm^−1^, the characteristic C−C stretching mode of the C_2_F_5_ group at 1340 cm^−1^. Weak and broad bands at 1187 and 1177 cm^−1^ in the IR and the Raman spectra (Figure S1.10 in the Supporting Information), respectively, are assigned to stretching modes of the CF_2_ group, and a strong IR absorption at 1105 cm^−1^ to the out‐of‐phase C−O stretching mode. The in‐phase C−O and C‐CF_2_ stretching modes of **2 c** are found in the Raman spectrum at 1132 and 1082 cm^−1^, respectively. The corresponding out‐of‐phase C‐CF_2_ stretching mode appeared in the IR spectrum at 1086 cm^−1^. Weak to medium intensity bands around 1000 cm^−1^ in both the IR and Raman spectra are due to CC_3_ stretching modes and a strong antisymmetric CF_2_ stretching mode is found in the IR spectrum at 898 cm^−1^ (calcd: 929 cm^−1^). The Raman active O−O stretching mode appears at 853 cm^−1^, in excellent agreement with the calculation at 852 cm^−1^, and also the C−O−O−C deformation of the peroxide, located at 352 cm^−1^ in the Raman spectrum, is very close to that of **2 b** (356 cm^−1^).

A full list of all experimental and computed wavenumbers together with tentative assignment is given in the Supporting Information, Table S1.2.

In previous studies, the synthetically valuable fluorinated radicals F_3_CO**^.^** or (F_3_C)_3_CO**^.^** were generated by photolysis of the corresponding peroxide (F_3_CO)_2_ (**2 a**) or [(F_3_C)_3_CO]_2_ (**2 b**), respectively.^19^ The recently recorded gas‐phase UV/Vis spectra of the perfluorinated bisalkyl peroxides (R^F^O)_2_ [R^F^=F_3_C **2 a**, (F_3_C)_3_C **2 b**, and (C_2_F_5_)(CF_3_)_2_C **2 c**] show that for **2 a** the lowest UV transition is below 200 nm, whereas the bulkier substituted peroxides **2 b**,**c** exhibit weaker redshifted transitions at 253 and 250 nm, respectively.^18^

As described previously, ferrocene, Fe^II^Cp_2_, is oxidized to ferrocenium, [Fe^III^Cp_2_]^+^, by addition of peroxide **2 b** [Eq. 6].^18^

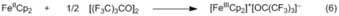



An immediate color change of the solid from orange to dark green is observed during the reaction, which is typical for the formation of a ferrocenium cation. Indeed, the IR spectrum of the solid (Figure S1.11 in the Supporting Information) shows the characteristic vibration modes of the ferrocenium cation.^44^ For example, the weak *ν*(CH) mode is blueshifted by 36 cm^−1^ to 3121 cm^−1^ with respect to ferrocene and the *ν*(CC) mode at 1421 cm^−1^ is also hypsochromically shifted by 14 cm^−1^ in comparison to the reactant ferrocene. The bands at 965, 724, and 536 cm^−1^ as well as strong absorption bands in the region from 1300 to 1100 cm^−1^ are characteristic for the anion, [OC(CF_3_)_3_]^−^.^45^ Additionally, the APCI mass spectra (Figure S1.12 in the Supporting Information) show a peak at *m*/*z*=235 in the negative mode, characteristic for the [OC(CF_3_)_3_]^−^ alkoxide anion. In the positive mode, an analogous decomposition pathway, compared to that of ferrocene, is observed. These spectra confirm the formation of [FeCp_2_][OC(CF_3_)_3_].

Elemental fluorine was added to a sample of [(F_3_C)_3_CO]_2_ (**2 b**) to a total pressure of about 1 bar at room temperature in a PFA tube. The tube was then flame‐sealed at liquid nitrogen temperatures and after reaching room temperature, NMR spectra were recorded (see the Supporting Information, Figure S1.13). The elemental fluorine is detected at a chemical shift of *δ*=425 ppm (liquid: 422±1 ppm, gaseous: 419±1 ppm).^46^ Peroxide **2 b** resists elemental fluorine, and its solubility in **2 b** is consistent with the low dipole moment as indicated by the dihedral angle *Θ* of the peroxide unit of 180° in the solid state and the perfluorinated nature of this compound.^18^ The longitudinal relaxation time *T*_1_ of the dissolved F_2_ was determined by an inversion recovery experiment to be *T*_1_=13.5 ms (Figure S1.13 in the Supporting Information). This is approximately 300 times larger than *T*_1_ for gaseous fluorine of 0.045 ms in the pressure range from 1 to 2 bar.^47^ This indicates that the fast relaxation owing to the spin‐rotation mechanism observed for gaseous fluorine is hindered and proves that the fluorine is indeed dissolved in peroxide **2 b**.

## Conclusion

We present a convenient synthesis to highly reactive perfluoro alkyl hypofluorite compounds R^F^OF from the corresponding alcohol and fluorine with excess CsF. Spectroscopic analysis of the hitherto undescribed (C_2_F_5_)(F_3_C)_2_COF with support of quantum‐chemical calculations are reported. We provide a new synthetic approach for perfluoro bisalkyl peroxides R^F^OOR^F^ by the reaction of hypofluorites with fluorinated silver wool. Furthermore, we also show the inertness of [(F_3_C)_3_CO]_2_ towards strong oxidizers such as elemental fluorine. The liquid temperature range from 16 to 99 °C for the nonpolar peroxide **2 b** together with its inertness towards strongly oxidizing halogens demonstrates its potential as a solvent for oxidation and halogenation reactions. By irradiation with UV light, perfluoro alkyl peroxides **2** can be activated to generate valuable R^F^O**^.^** radicals for synthetic applications such as perfluoro alkoxy group transfer reagents.

## Experimental Section

Experiments were carried out under strictly dry and oxygen‐free conditions in glass tubes with Teflon valves or in stainless‐steel vessels. Purchased starting materials were used without further purification. NMR spectra of neat liquid substances were recorded with a JEOL 400 MHz ECS or ECZ spectrometer by using a capillary filled with [D_6_]acetone (^1^H NMR: 2.05 ppm, 400.53 MHz; ^13^C NMR: 29.8 ppm, 100.51 MHz) and CFCl_3_ (^19^F NMR: 0 ppm, 376.13 MHz) as external standards. The chemical shift and scalar coupling constants were obtained by the program Mestrenova 10.0.^48^ Raman spectra were measured at liquid nitrogen temperature with a Bruker MultiRAM II spectrometer equipped with a 1064 nm CW DPSS laser and a LN_2_ cooled germanium detector at a resolution of 4 cm^−1^. Gas‐phase infrared spectra were recorded by using a Bruker Vector 22 spectrometer at a resolution of 2 cm^−1^. UV/Vis spectra of gaseous samples were obtained by using a PerkinElmer Lambda‐900 spectrophotometer. Mass spectra were measured with an Advion expression^L^ compact mass spectrometer. The *m*/*z* values of the monoisotopic peaks are given. The NMR relaxation time *T*_1_ was determined by the 180°–*τ*–90° pulse sequence technique. Powder diffraction data were collected with a STOE IPDS II/T instrument at 290 K with Mo_Kα_ radiation (*λ*=0.71073 Å) by using a graphite monochromator. Integration was performed with STOE X‐Area V1.56, data analysis and Rietveld refinement were performed with X′Pert HighScore Plus V2.2c.

### Safety note

***Caution***! Extreme caution should be exercised when working with elemental fluorine and hypofluorites. Explosions have been reported^2^, ^49^ during handling of these extremely hazardous compounds. Although the described perfluoroalkyl peroxides were found to be insensitive to shock and friction^18^ according to the U.N. Recommendations on the Transport of Dangerous Goods,^50^ we cannot exclude explosive reactions in mixtures with other substances.

## Conflict of interest

The authors declare no conflict of interest.

## Supporting information

As a service to our authors and readers, this journal provides supporting information supplied by the authors. Such materials are peer reviewed and may be re‐organized for online delivery, but are not copy‐edited or typeset. Technical support issues arising from supporting information (other than missing files) should be addressed to the authors.

Supplementary
