# Supplementary material for: Perfluoro Alkyl Hypofluorites and Peroxides Revisited
Source: Chemistry. 2019 Oct 23;25(64):14721–7. doi: 10.1002/chem.201903620 (PMC6899984; doi:10.1002/chem.201903620)
Supplement: Supplementary file 1 — Supplementary [file CHEM-25-14721-s001.pdf]

CHEMISTRY
A European Journal

Supporting Information

Perfluoro Alkyl Hypofluorites and Peroxides Revisited

Jan H. Nissen, Thomas Drews, Benjamin Schröder, Helmut Beckers, Simon Steinhauer, and Sebastian Riedel*[a]

chem_201903620_sm_miscellaneous_information.pdf

Table of Contents

1. **Experimental Details** 2

 1.1 **Fluorination of Silver Wool** 2

 1.2 **Synthesis of Perfluoro 2-methyl 2-butanol** 2

 1.3 **Syntheses of Hypofluorites** 2

 1.4 **Syntheses of Peroxides** 4

 1.5 **Ferrocenium nonafluoro-*tert*-butoxide** 6

 1.6 **XRD, MS, NMR and Vibrational Spectra** 6

2. **Computational Details** 13

 2.1 **$(C_2F_5)(F_3C)_2COF$ – Rotational Isomers** 13

3. **References** 15

1. Experimental Details

Experiments were carried out at strictly dry and oxygen free conditions in glass tubes using Teflon® valves or in stainless steel vessels. Purchased starting material was used without further purification. NMR spectra of neat liquid substances were recorded on a JEOL 400 MHz ECS or ECZ spectrometer using a capillary filled with [D6]Acetone. Reported chemical shifts are referenced to the Ξ values given in IUPAC recommendations of 2008[1] using the 2H signal of the deuterated solvent as internal reference. For external locking [D6]Acetone was flame sealed in a glass capillary and the lock oscillator frequency was adjusted to give δ(1H) = 7.26 ppm for a $CHCl_3$ sample locked on the capillary. The chemical shift and scalar coupling constants were obtained by the program Mestrenova 10.0.[2] Raman spectra were measured at liquid nitrogen temperature on a Bruker MultiRAM II spectrometer equipped with a 1064 nm CW DPSS laser and a LN_2 cooled germanium detector at a resolution of 4 cm^{-1}. Gas-phase infrared spectra were recorded using a Bruker Vector 22 spectrometer at a resolution of 2 cm^{-1}. UV/Vis-spectra of gaseous samples were obtained using a Perkin-Elmer Lambda-900 spectro-photometer. Mass spectra were measured with an Advion expression^L compact mass spectrometer. The m/z values of the monisotopic peaks are given. The NMR relaxation time T_1 was determined by the inversion recovery experiment. The sample of **2b** with elemental F_2 was flame sealed in a PFA-tube (outer diameter 3.8 mm, inner diameter 2.6 mm). The PFA-tube was then inserted into a 5 mm NMR tube filled with a small amount of [D6]Acetone. Powder diffraction data were collected on a STOE IPDS II/T instrument at 290 K with Mo K_α radiation (λ = 0.71073 Å) using a graphite monochromator. Integration was performed with STOE X-Area V1.56, data analysis and Rietveld refinement was performed with X'Pert HighScore Plus V2.2c.

Safety note: Extreme caution should be exercised when working with elemental fluorine and hypofluorites. Explosions have been reported[3,4] handling these extremely hazardous compounds. Although the described perfluoroalkyl peroxides were found to be insensitive to shock and friction[5] according to the U. N. Recommendations on the Transport of Dangerous goods,[6] we cannot exclude explosive reactions in mixtures with other substances.

1.1 Fluorination of Silver Wool

Elemental silver wool (5 g) was treated with small portions of fluorine at room temperature in a stainless steel reactor (V = 720 mL) until the pressure remains constant. Afterwards the vessel was heated to 100 °C and further fed with fluorine until no consumption of fluorine was observed. Excess fluorine was then removed by evacuating the reactor through a tube filled with Soda Lime, as prior described.[5]

1.2 Synthesis of Perfluoro 2-methyl 2-butanol

According to a descriped syntheses[7] a Schlenk flask was equipped with dryed KF (5.22 g, 89.8 mmol) and acetonitrile (~20 mL) was added at liquid nitrogen temperatures. Afterwards octafluoro-2-butanone (75 mmol, 1 eq.) and trimethyl(trifluoromethyl)silane (82 mmol, 1.1 eq.) were enclosed by condensation. At –40 °C the flask was filled with argon and the reaction mixture was stirred for 1 h. Equipped with a bubbler the flask was then allowed to reach room temperature giving a slightly brownish solution. After additional stirring for 6 h all volatiles were removed under reduced pressure and the remaining residue was cooled with ice and concentrated H_2SO_4 (25 mL) was added dropwise. The volatile compounds were collected by pumping through a trap cooled to –196 °C containing the crude product. Perfluoro 2-methyl 2-buthanol was purified by trap-to-trap distillation (–40, –90, –196 °C) in dynamic vacuum and kept at –90 °C as a colorless solid (18.43 g, 64 mmol, 86%).

^{1}H NMR (neat, external [D6]Acetone, r.t.): δ [ppm] = 5.67 (s).

^{13}C {^{19}F} DEPTQ NMR (neat, external [D6]Acetone, r.t.): δ [ppm] = 120.7 (C(CF$_3$)$_2$), 117.8 (CF_3), 111.6 (CF_2), 78.4 (C_q).

^{19}F NMR (neat, external [D6]Acetone, r.t.): δ [ppm] = –73.9 (tq, 6F, ^4J(F,F) = 11.6 Hz, ^5J(F,F) = 5.8 Hz, C(CF_3)$_2$), –81.5 (sept, 3F, ^5J(F,F) = 5.9 Hz, CF_3), –120.1 (sept, 2F, ^4J(F,F) = 11.3 Hz, CF_2).

IR (gas): $\tilde{\nu}$ [cm^{-1}] = 3629 (s), 1377 (w), 1342 (w), 1278 (vs), 1250 (vs), 1230 (vs), 1204 (sh), 1145 (s), 1073 (m), 968 (s), 953 (sh), 882 (s), 738 (s), 725 (s), 621 (w), 538 (w).

1.3 Syntheses of Hypofluorites

F_3COF, CF_3CF_2OF and $(F_3C)_2CFOF$: This hypofluorites were prepared from the reaction of elemental fluorine and the corresponding cabonyl compound [F_2CO, $F_3CC(O)F$, $(F_3C)_2CO$] as described in the literature.[3]

$(F_3C)_3COF$: Freshly destilled perfluoro *tert*-butanol (1.42 g, 6 mmol) was added to dry CsF (50 g) in a stainless steel vessel (V = 720 mL) by condensation at –196 °C and then the reactor was warmed to room temperature while shaking. Elemental fluorine was then added in small portions at –78 °C (for safety reasons only solid CO_2 without liquid refrigerant should be used). When no further consumption of fluorine was observed the temperature was held at –78 °C for another 20 min.

Afterwards the excess of fluorine was removed at –196 °C. Pure perfluoro *tert*-butyl hypofluorite (1.47 g, 5.8 mmol, 97%) retained in a –110 °C trap by trap-to-trap distillation.

^{13}C NMR (neat, external [D6]Acetone, r.t.): δ [ppm] = 118.8 (CF_3), 87.0 (C_q).

^{19}F NMR (neat, external [D6]Acetone, r.t.): δ [ppm] = 148.4 (decet, 1F, ^4J(F,F) = 16.4 Hz, OF), –69.7 (d, 9F, ^4J(F,F) = 16.4 Hz, CF_3).

IR (gas): $\tilde{\nu}$ [cm^{-1}] = 1312 (s), 1291 (vs), 1270 (s), 1265 (s), 1239 (w), 1192 (w), 1108 (m), 1015 (m), 984 (m), 888 (w), 741 (m), 736 (m), 731 (m), 540 (w), 503 (w).

APCI$^-$: m/z = 285 (7.9%) $[(F_3C)_3COCF_2]^-$, 235 (100.0%) $[(F_3C)_3CO]^-$, 185 (2.3%) $[(F_3C)_2CFO]^-$, 183 (3.3%) $[C_3F_7N]^-$, 113 (4.3%) $[CF_3N_2O]^-$, 69 (2.6%) $[CF_3]^-$.

APCI$^+$: m/z = 284 (33.8%) $[(F_3C)_3CONFO]^+$, 256 (100.0%) $[C_3F_{10}NO]^+$, 149 (7.8%) $[C_2F_5NO]^+$, 118 (1.6%) $[CF_3ONF]^+$, 90 (3.6%) $[C_3F_2O]^+$, 72 (5.3%) $[C_2FNO]^+$, 45 (5.6%) $[CFN]^+$.

$(C_2F_5)(F_3C)_2COF$: Perfluoro 2-methyl-2-butanol (1.72 g, 6 mmol) was distilled onto CsF (50 g) in a stainless steel reactor (V = 720 mL) –196 °C and then the reactor warmed to room temperature. After shaking, the reactor was cooled to –78 °C and elemental fluorine was added in small portions until no further fluorine was consumed. The temperature was kept at –78 °C for another 20 min. Excess fluorine was removed at liqiud nitrogen temperatures by evacuating the reactor through a tower filled with Soda Lime. Trap-to-trap distillation yields pure perfluoro (2-methyl-2-butyl) hypofluorite (1.58 g, 5.2 mmol, 87%) which retained in a –110 °C trap as a colorless solid.

^{13}C NMR (neat, external [D6]Acetone, –60 °C): δ [ppm] = 118.5 (qd, ^1J(F,C) = 293 Hz, ^2J(F,C) = 9 Hz, C(CF_3)$_2$,), 116.8 (qtd, ^1J(F,C) = 289 Hz, ^2J(F,C) = 31 Hz, ^4J(F,C) = 19 Hz, CF_3), 109.8 (tm, ^1J(F,C) = 274 Hz, CF_2), 87.7 (m, C_q).

^{19}F NMR (neat, external [D6]Acetone, –60 °C): δ [ppm] = 150.8 (sept t q, 1F, ^4J(F,CF_3) = 17.7 Hz, ^4J(F,CF_2) = 11.6 Hz, ^5J(F,CF_3) = 5.8 Hz, O*F*), –68.3 (dtq, 6F, ^4J(F,OF) = 17.7 Hz, ^4J(F,CF_2) = 11.6 Hz, ^5J(F,CF_3) = 5.8 Hz, C(CF_3)$_2$), –81.9 (dsept, 3F, ^5J(F,OF) = 5.8 Hz, ^5J(F,CF_3) = 5.8 Hz, CF_3), –117.8 (dsept, 2F, ^4J(F,OF) = 11.6 Hz, ^4J(F,F) = 11.6 Hz, CF_2).

IR (gas): $\tilde{\nu}$ [cm^{-1}] = 1342 (m) 1291 (s,sh) 1276 (vs) 1262 (vs), 1255 (vs), 1251 (vs), 1232 (s), 1181 (m), 1161 (w, sh), 1107 (m), 1078 (w), 1038 (vw), 1003 (m), 977 (w), 925 (w), 913 (w), 895 (m), 878 (m), 766 (vw), 743 (m), 730 (m), 613 (vw), 558 (sh), 539 (vw), 513 (vw) 484 (vw).

Table S1.1. Gas-phase vibrational frequencies $\tilde{\nu}$ [cm^{-1}] and relative IR band intensities[a] compared to computed values for different rotational conformers of $(C_2F_5)(F_3C)_2COF$ (**1c**) at the B3LYP/aug-cc-pVTZ level of theory.[b]

experiment	DFT						assignment
	t-1	*t*-2	*t*-2‘	*g*-1	*g*-2	*g*-2‘	
1342 (m)	1290 (75)	1292 (47)	1296 (42)	1308 (22)	1302 (52)	1301 (54)	ν(F_2C–CF_3)
1291 (s,sh)	1273 (272)	1261 (375)	1256 (494)	1260 (421)	1264 (416)	1264 (415)	ν(CF_3)
1276 (vs)	1248 (606)	1252 (597)	1251 (395)	1246 (541)	1248 (314)	1247 (348)	ν(CF_3)
1262 (vs)	1239 (403)	1248 (287)	1244 (418)	1237 (338)	1240 (417)	1239 (383)	ν(CF_3)
1255 (vs)	1233 (58)	1222 (48)	1222 (87)	1221 (157)	1222 (42)	1221 (38)	ν(CF_3)
1251 (vs)	1218 (110)	1217 (182)	1221 (84)	1216 (240)	1206 (259)	1205 (275)	ν(CF_3)
1232 (s)	1197 (297)	1201 (224)	1201 (268)	1201 (256)	1203 (385)	1202 (362)	ν(CF_3)
1181 (m)	1161 (25)	1165 (105)	1160 (105)	1155 (5)	1164 (40)	1166 (46)	ν(CF_3)
1161 (w, sh)	1154 (63)	1155 (20)	1155 (20)	1153 (11)	1155 (18)	1154 (17)	ν(CF_2)
1107 (m), 1078 (w)	1064 (121)	1081 (29)	1083 (34)	1113 (14)	1093 (54)	1092 (54)	ν(CO)
1038 (vw)	1055 (12)	1023 (96)	1024 (109)	1047 (49)	1055 (66)	1054 (65)	ν(C–CF_2)
1003 (m), 977 (w)	972 (100)	1014 (44)	1011 (29)	963 (93)	981 (41)	981 (40)	ν_{as}(C–$(CF_3)_2$)
925 (w), 913 (w)	995 (25)	951 (78)	952 (74)	967 (34)	960 (76)	960 (77)	ν(OF)
895 (m), 878 (m)	888 (117)	924 (137)	923 (137)	898 (150)	906 (120)	905 (119)	ν(F_2C–CF_3)
766 (vw)	766 (5)	761 (8)	760 (8)	765 (1)	759 (4)	758 (4)	δ(CF_3)
743 (m)	739 (59)	739 (44)	739 (38)	740 (59)	735 (51)	735 (47)	δ(CF_3)
730 (m)	725 (39)	734 (42)	733 (48)	725 (40)	734 (42)	734 (46)	δ($C(CF_3)_2$)
613 (vw)	619 (6)	599 (3)	601 (4)	621 (12)	616 (11)	616 (11)	δ(CC_3)
558 (sh)	537 (4)	540 (2)	541 (2)	537 (3)	538 (5)	537 (5)	δ(CF_3)
539 (vw)	525 (9)	531 (11)	532 (10)	527 (10)	537 (1)	536 (1)	δ(CF_3)
513 (vw)	501 (7)	492 (10)	493 (10)	499 (8)	504 (9)	503 (9)	δ(CF_3)
484 (vw)	444 (2)	445 (1)	440 (1)	445 (3)	436 (3)	435 (3)	δ(C_2F_5)

[a] Relative intensities in parentheses: vw = very weak, w = weak, m = medium, s = strong, vs = very strong, sh = shoulder.
[b] For the different *trans* and *gauche* rotational conformers see Figure 3 and Figures S2.

1.4 Syntheses of Peroxides

$(F_3CO)_2$: Bis(trifluoromethyl) peroxide was synthesized as descriped in the literature.[8]
^{13}C NMR (neat, external [D6]Acetone, r.t.): δ [ppm] = 122.9 (q, ^{1}J(F,C) = 267.7 Hz).
^{17}O NMR (54.19 MHz, neat, external [D6]Acetone, r.t.): δ [ppm] = 262 (s).
^{19}F NMR (neat, external [D6]Acetone, r.t.): δ [ppm] = –72.3 (s).

$[(F_3C)_3CO]_2$: Perfluoro *tert*-butyl hypofluorite (2.0 mmol) was transferred in a steel vessel containing fluorinated silver wool. After three days at –48 °C the desired perfluoro bis(*tert*-butyl) peroxide (0.72 mmol, 72%) was obtained and purified by trap-to-trap distillation at –78 °C.
^{13}C {^{19}F} DEPTQ NMR (neat, external [D6]Acetone, r.t.): δ [ppm] = 118.8 (CF_3), 84.3 (C_q).
^{17}O NMR (54.19 MHz, neat, external [D6]Acetone, r.t.): δ [ppm] = 246 (s).
^{19}F NMR (neat, external [D6]Acetone, r.t.) : δ [ppm] = –69.6 (s).
IR: $\tilde{\nu}$ [cm^{-1}] = 1311 (s, sh), 1302 (s, sh), 1288 (vs), 1259 (s), 1226 (w), 1110 (s), 1002 (m), 982 (m), 772 (vw), 739 (m), 731 (m), 541 (w), 496 (w).
Raman: $\tilde{\nu}$ [cm^{-1}] = 1311 (m, sh), 1294 (m), 1278 (m, sh), 1129 (s), 1027 (s), 872 (m, sh), 865 (s), 783 (vs), 771 (w), 749 (s), 691(vw), 569 (w), 545 (m, sh), 541 (m), 523 (w), 356 (m), 339 (m), 326 (s, sh), 319 (s), 305 (m), 296 (w), 266 (w), 260 (w, sh), 241 (s), 194 (w), 123 (s), 118 (m, sh).
APCI^{-}: m/z = 235 (100.0%) $[(F_3C)_3CO]^-$, 219 (75.9%) $[(F_3C)_3C]^-$, 185 (97.7%) $[(F_3C)_2CFO]^-$, 69 (16.8%) $[CF_3]^-$.

$[(C_2F_5)(F_3C)_2CO]_2$: Perfluoro 2-methyl-2-butyl hypofluorite (2.0 mmol) was distilled into a stainless steel reactor containing fluorinated silver wool at –196 °C. The reactor was held at –48 °C for 72 h. The volatile reaction products were distilled out of the reactor and perfluoro bis(2-methyl-2-butyl) peroxide was obtained by trap-to-trap distillation in a –78 °C trap as a colorless solid (0.7 mmol, 66%).
^{13}C (^{19}F) DEPTQ NMR (neat, r.t.): δ [ppm] = 118.5 ($C(CF_3)_2$), 116.7 ($\mathit{C}F_3$), 115.2 ($\mathit{C}F_2$), 85.6 ($\mathit{C}C_3$).
^{19}F NMR ($CFCl_3$, r.t.): –67.0 (m, 12F, $C(CF_3)_2$), –80.1 (m, 6F, CF_3), –115.6 (sept, 4F, ^{4}J(F,F) = 11.5 Hz, CF_2).
IR (gas): $\tilde{\nu}$ [cm^{-1}] = 1340 (w), 1290 (s), 1277 (s, sh), 1269 (s), 1254 (vs), 1229 (s), 1187 (w), 1177 (w, sh), 1105 (s), 1086 (m), 1077 (m, sh), 1009 (w), 986 (m), 973 (m), 898 (s), 766 (w), 743 (s), 730 (s), 697 (vw), 656 (vw), 631 (w), 542 (w), 511 (w), 447 (vw).
Raman: $\tilde{\nu}$ [cm^{-1}] = 1342 (m) 1289 (w) 1277 (s) 1245 (s), 1238 (w, sh) 1177 (vw) 1132 (m), 1125 (m, sh) 1082 (m) 1009 (w), 997 (w), 853 (s), 781 (vs), 766 (m), 751 (vs), 731 (vw), 686 (w), 657 (w), 632 (m), 596 (m), 566 (m, sh), 554 (m), 548 (m), 532 (mw), 449 (w), 373 (m), 352 (m), 332 (s), 323 (m, sh), 309 (m), 295 (s), 261 (w), 243 (s), 232 (m, sh), 199 (w), 117 (s).

Table S1.2. Vibrational frequencies $\tilde{\nu}$ [cm^{-1}][a] of $[(F_3C)_3CO]_2$ (**2b**) and $[(C_2F_5)(F_3C)_2CO]_2$ (**2c**) compared to quantum chemical calculations (B3LYP/aug-cc-pVTZ) with assignment to the corresponding normal modes.

$[(F_3C)_3CO]_2$ (**2b**)			$[(C_2F_5)(F_3C)_2CO]_2$ (**2c**)			
experiment		calc. (IR/Raman)	experiment		calc. (IR/Raman)	assignment
IR	Raman		IR	Raman		
				1342 (m)	1294 (34/12)	$\nu_{i.p.}(F_2C–CF_3)$
			1340 (w)		1285 (86/0)	$\nu_{o.p.}(F_2C–CF_3)$
				1289 (w)	1272 (145/6)	$\nu_{i.p.}(C(CF_3)_2)$
			1290 (s)		1271 (716/1)	$\nu_{o.p.}(C(CF_3)_2)$
1311 (s, sh)	1311 (m, sh)	1277 (66/7)				$\nu(CF_3)$
			1277 (s, sh)	1277 (s)	1249 (126/4)	
1302 (s, sh)		1272 (504/1)				
			1269 (s)		1246 (705/0)	
	1294 (m)	1270 (88/10)				
			1254 (vs)		1245 (875/2)	
1288 (vs)		1263 (800/2)				
				1245 (s)	1237 (315/4)	
	1278 (m, sh)	1250 (226/5)				
				1238 (w, sh)	1236 (0/3)	
1259 (s)		1235 (461/0)				
			1229 (s)		1197 (561/1)	
1226 (w)		1195 (42/0)				
			1187 (w)		1184 (38/0)	$\nu(CF_2)$
			1177 (w, sh)	1177 (vw)	1183 (17/1)	
	1129 (s)	1113 (14/54)		1132 (m), 1125 (m, sh)	1088 (4/49)	$\nu_{i.p}(CO)$
1110 (s)		1099 (487/1)	1105 (s)		1076 (164/1)	$\nu_{o.p}(CO)$
				1082 (m)	1057 (0/7)	$\nu(C–CF_2)$
			1086 (m), 1077 (m, sh)		1037 (242/0)	$\nu(C–CF_2)$
	1027 (s)	1028 (2/53)				$\nu(CC_3)$
			1009 (w)	1009 (w)	988 (59/100)	
1002 (m)		994 (235/0)				
			986 (m), 973 (m)	997 (w)	969 (119/40)	
982 (m)		970 (218/0)				
			898 (s)		929 (330/0)	$\nu(CF_2)$
	872 (m, sh), 865 (s)	902 (0/100)		853 (s)	852 (1/59)	$\nu(OO)$
	783 (vs)	778 (0/51)		781 (vs)	778 (0/24)	$\delta(CF_3)$
772 (vw)	771 (w)	766 (6/0)	766 (w)	766 (m)	762 (25/0)	
	749 (s)	744 (1/48)		751 (vs)	741 (1/29)	
739 (m)		725 (68/0)	743 (s)		740 (102/0)	
731 (m)		724 (4/0)	730 (s)	731 (vw)	725 (72/3)	
	691(vw)	685 (0/5)	697 (vw)	686 (w)	694 (2/8)	$\delta_s(CC_3)$
			656 (vw)	657 (w)	654 (3/1)	$\delta(C_2F_5)$
			631 (w)	632 (m)	629 (2/4)	
				596 (m)	590 (0/9)	
	569 (w)	571 (0/4)		566 (m, sh)	561 (0/2)	$\delta(CF_3)$
				554 (m)	545 (0/7)	
	545 (m, sh)	534 (7/5)		548 (m)	546 (0/11)	
541 (w)	541 (m)	533 (9/6)	542 (w)		531 (18/0)	
	523 (w)	516 (0/10)		532 (mw)	525 (1/10)	
496 (w)		487 (15/0)	511 (w)		486 (22/1)	
			447 (vw)	449 (w)	444 (1/3)	$\delta(C_2F_5)$
				373 (m)	365 (0/14)	
	356 (m)	354 (0/19)		352 (m)	350 (1/18)	$\delta(COOC)$
	339 (m)	332 (0/17)		332 (s)	335 (0/14)	$\rho(CF_3)$
	326 (s, sh)	316 (0/26)		323 (m, sh)	318 (0/19)	
	319 (s)	310 (0/47)				
	305 (m)	298 (0/26)		309 (m)	308 (0/27)	
	296 (w)	289 (0/8)		295 (s)	287 (0/25)	
	266 (w)	268 (2/4)		261 (w)	260 (0/5)	$\delta(CC_3)$
	260 (w, sh)	257 (0/3)				
	241 (s)	236 (0/79)		243 (s)	230 (4/32)	
				232 (m, sh)	222 (1/13)	$\delta(C_2F_5)$
	194 (w)	184 (0/7)		199 (w)	187 (0/15)	$\delta(CC_3)$
	123 (s)	120 (0/46)		117 (s)	103 (0/65)	
	118 (m, sh)	112 (0/18)				

[a] Relative intensities in parentheses: vw = very weak, w = weak, m = medium, s = strong, vs = very strong, sh = shoulder.

1.5 Ferrocenium nonafluoro-*tert*-butoxide

Ferrocene (19 mg, 0.1 mmol) was filled in a Schlenk flask equipped with a teflon stopcock and bis(nonafluoro-*tert*-butyl) peroxide (0.5 mmol) was added at LN_2 temperatures by distillation. When the mixture was warmed to room temperature the color of the solid changed from orange to dark green. Excess of bis(nonafluoro-*tert*-butyl) peroxide was then removed under a reduced pressure and ferrocenium nonafluoro-*tert*-butoxide was obtained as a dark green solid (48 mg, 0.1 mmol, 99%).

IR (ATR): $\tilde{\nu}$ [cm^{-1}] = 3121 (w), 1686 (w), 1421 (w), 1408 (sh), 1323 (sh), 1297 (sh), 1264 (sh), 1234 (vs), 1182 (vs), 1159 (sh), 1104 (m), 1013 (sh), 1000 (m), 965 (vs), 881 (w), 881 (w), 854 (s), 812 (m), 784 (m), 724 (vs), 630 (w), 570 (w), 536 (s), 487 (sh), 472 (s).

APCI$^-$: m/z = 235 (100.0%) $[(F_3C)_3CO]^-$, 185 (4.6%) $[(F_3C)_2CFO]^-$.

APCI$^+$: m/z = 149 (%), 118 (%), 90 (100%), 72 (%), 56 (%).

1.6 XRD, MS, NMR and Vibrational Spectra

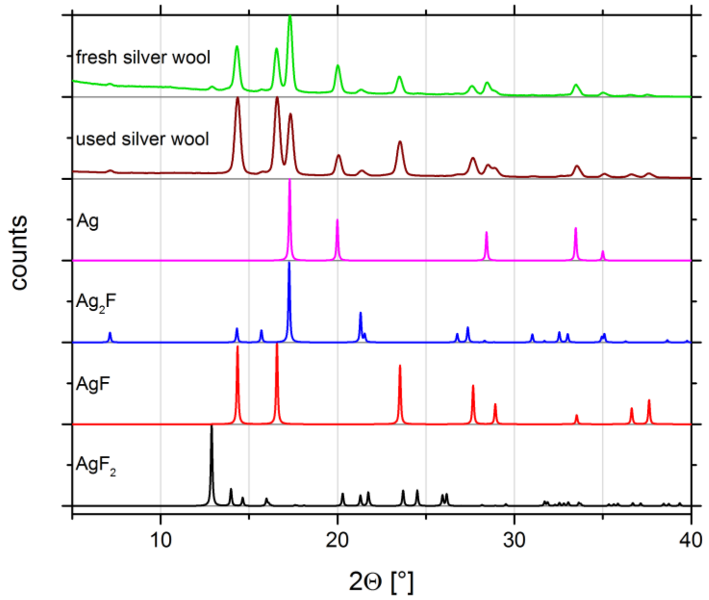

Figure S1.1. Powder X-ray diffraction patterns of fluorinated silver wool prior (green) and after (brown) several reactions in comparison to that of Ag (*Fm*$\bar{3}$*m*, purple) [9], Ag_2F (*P*$\bar{3}$*m*, blue)[10], AgF (*Fm*$\bar{3}$*m*, red)[11] and AgF_2 (*Pbca*, black)[12].

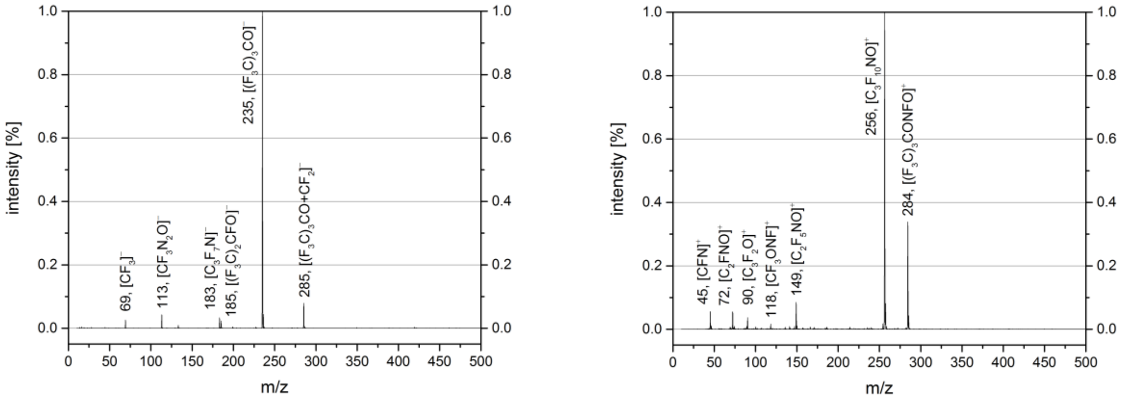

Figure S1.2. APCI mass spectra of $(F_3C)_3COF$ (**1b**) in the negative (left) and positive mode (right).

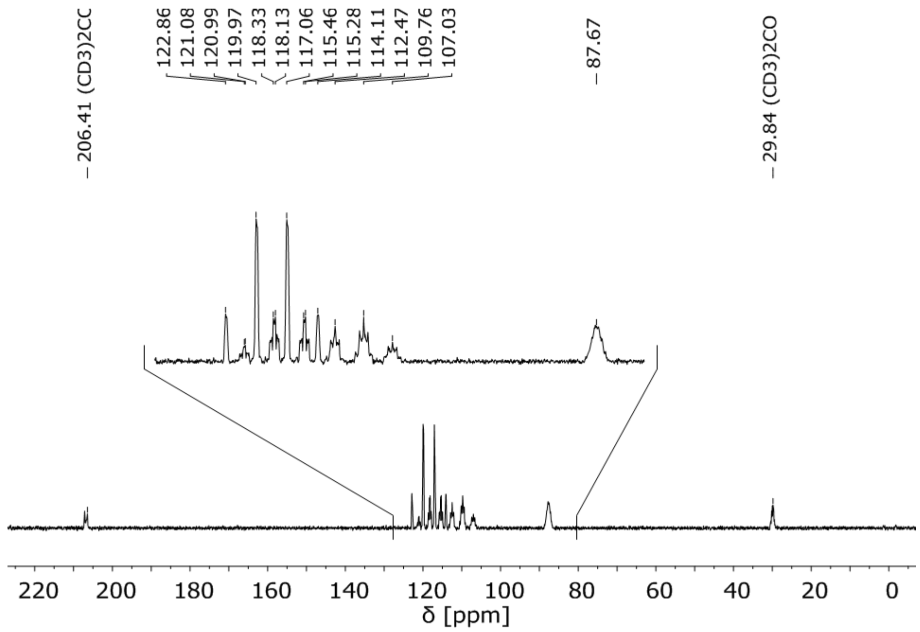

Figure S1.3. ^{19}F coupled ^{13}C NMR spectrum of $(C_2F_5)(F_3C)_2COF$ (**1c**) with partial enlarged details (101 MHz, neat, external [D6]Acetone, –60 °C).

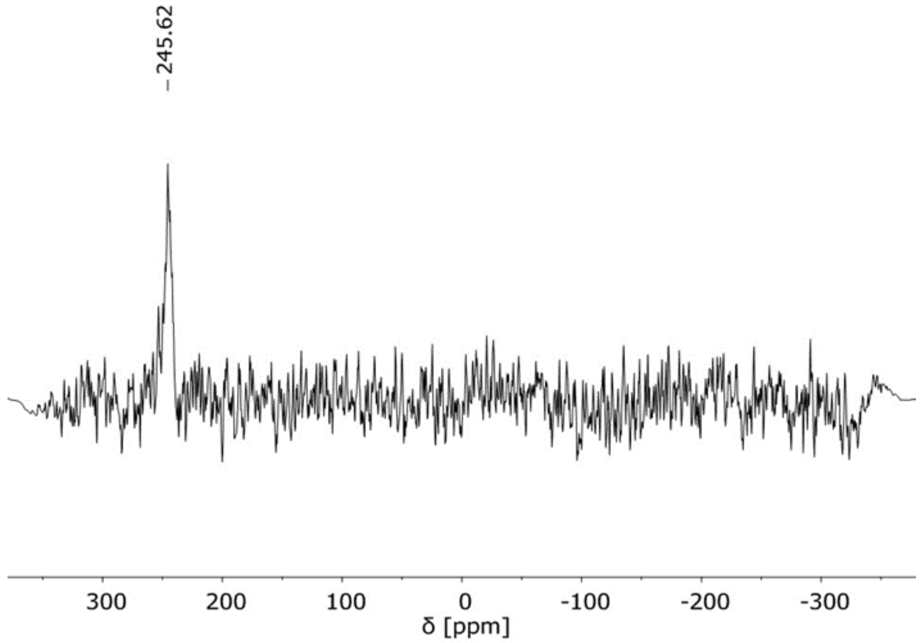

Figure S1.4. ^{17}O NMR spectrum of $[(F_3C)_3CO]_2$ (**2b**) (54.19 MHz, neat, external [D6]Acetone, r.t.).

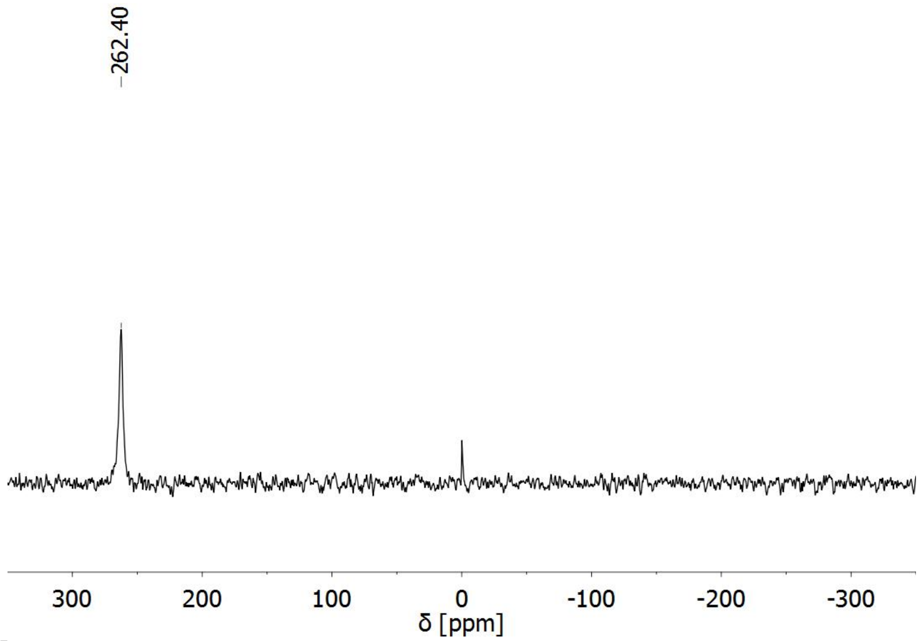

Figure S1.5. ^{17}O NMR spectrum of $(F_3CO)_2$ (**2a**) (54.19 MHz, neat, external [D6]Acetone, r.t.).

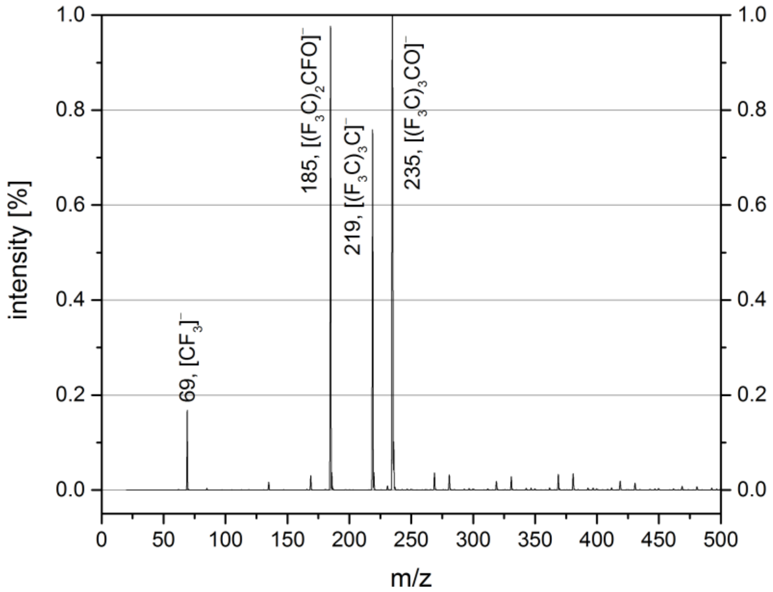

Figure S1.6. APCI mass spectrum of $[(F_3C)_3CO]_2$ (**2b**) in the negative mode.

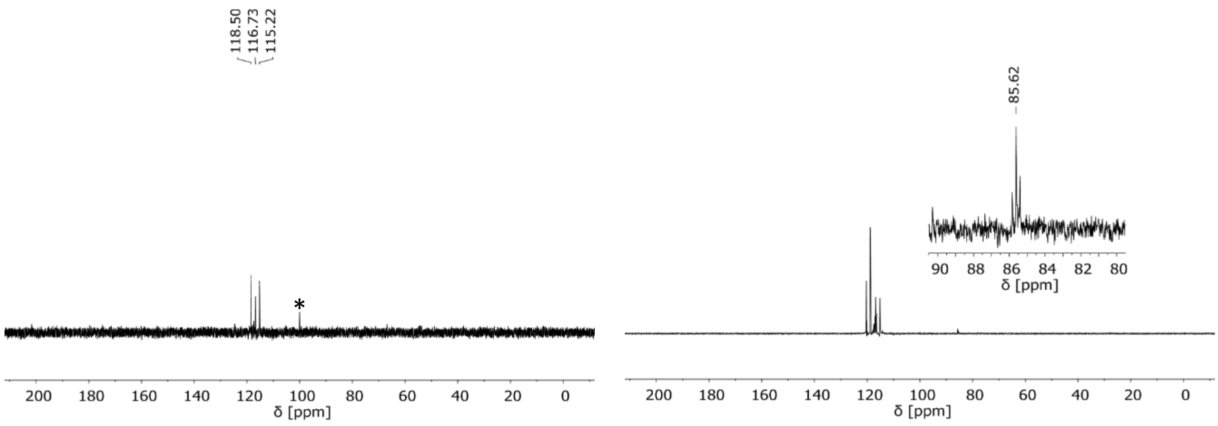

Figure 1.7. ^{13}C {^{19}F} DEPTQ NMR spectra of $[(C_2F_5)(F_3C)_2CO]_2$ (**1c**) with an optimized ^{1}J coupling constant of 290 Hz (left) and a ^{2}J coupling constant of 35 Hz (right) (101 MHz, *n*-pentane, external [D6]Acetone, r.t.). The left spectrum shows the spectrometer frequency at 100.0 ppm (*) while the ^{2}J optimized spectrum possesses coupling artefacts for the fluorine bonded carbon atoms around 120 ppm. The quartanary carbon atom resonates at 85.6 ppm.

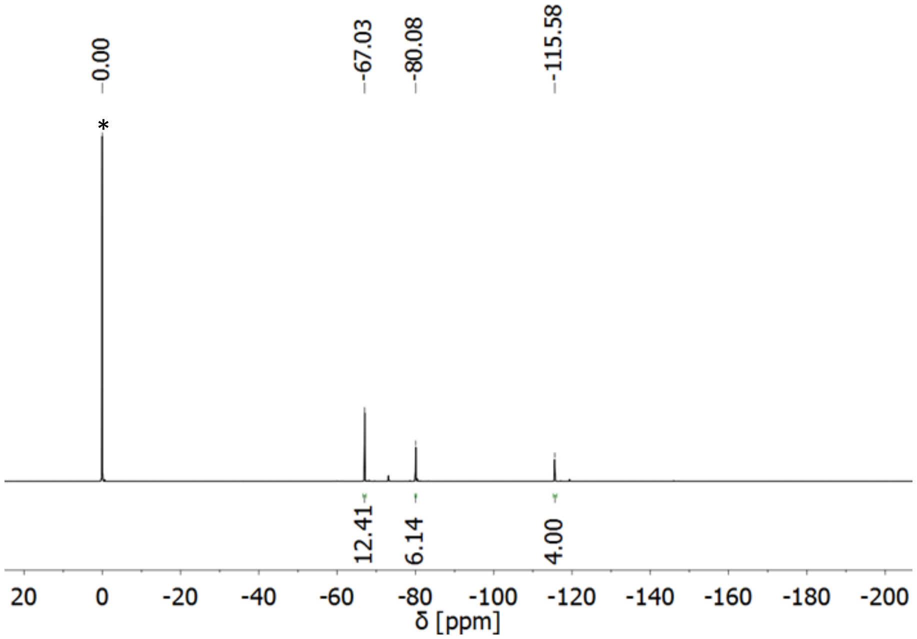

Figure 1.8. ^{19}F NMR spectrum of $[(C_2F_5)(F_3C)_2CO]_2$ (**1c**) (376.13 MHz, $CFCl_3$(*), r.t.).

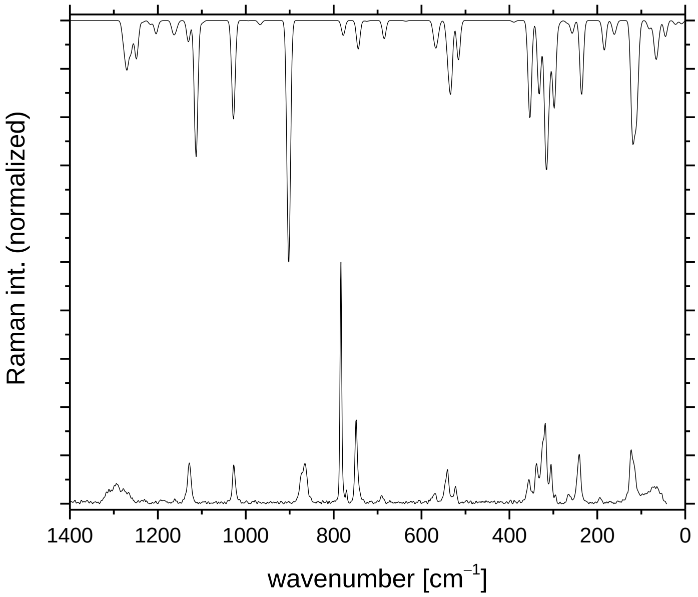

Figure S1.9. Raman spectrum of $[(F_3C)_3CO]_2$ (**2b**) in the solid state (bottom) in comparison to the computed spectrum at the B3LYP/aug-cc-pVTZ level of theory at the top trace.

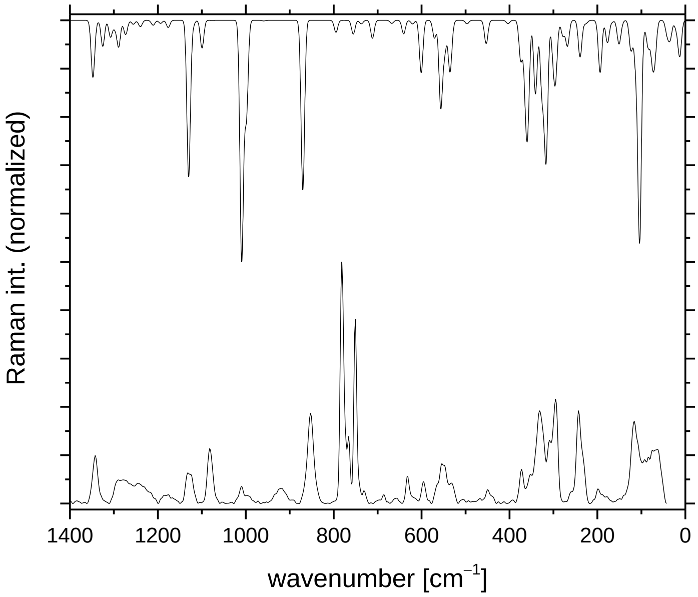

Figure S1.10. Raman spectrum of $[(C_2F_5)(F_3C)_2CO]_2$ (**2c**) in the solid state (bottom) in comparison to the computed spectrum at the B3LYP/aug-cc-pVTZ level of theory at the top trace.

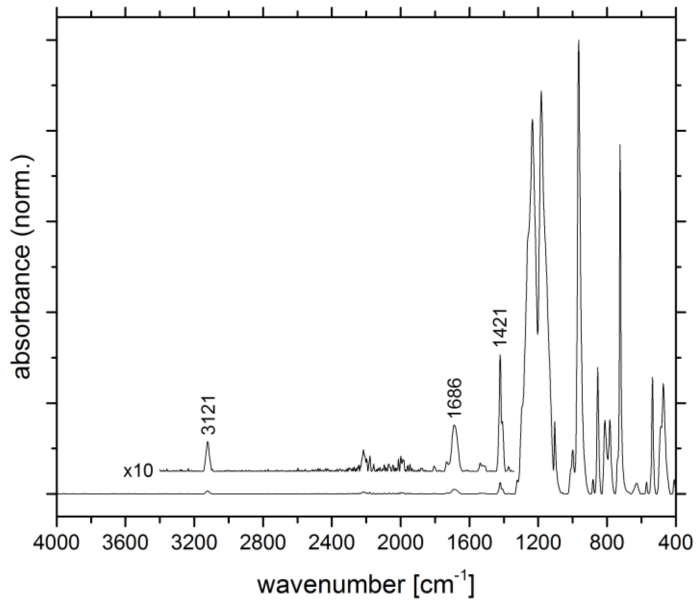

Figure S1.11. ATR IR spectrum of $[FeCp_2][OC(CF_3)_3]$ with partial enlargement.

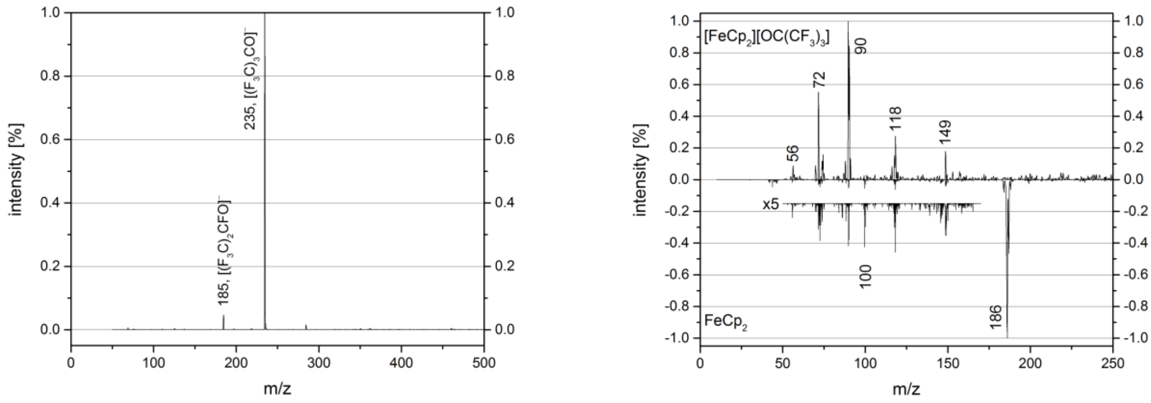

Figure S1.12. APCI mass spectra of $[FeCp_2][OC(CF_3)_3]$ in the negative (left) and positive mode (right, in comparison to ferrocene).

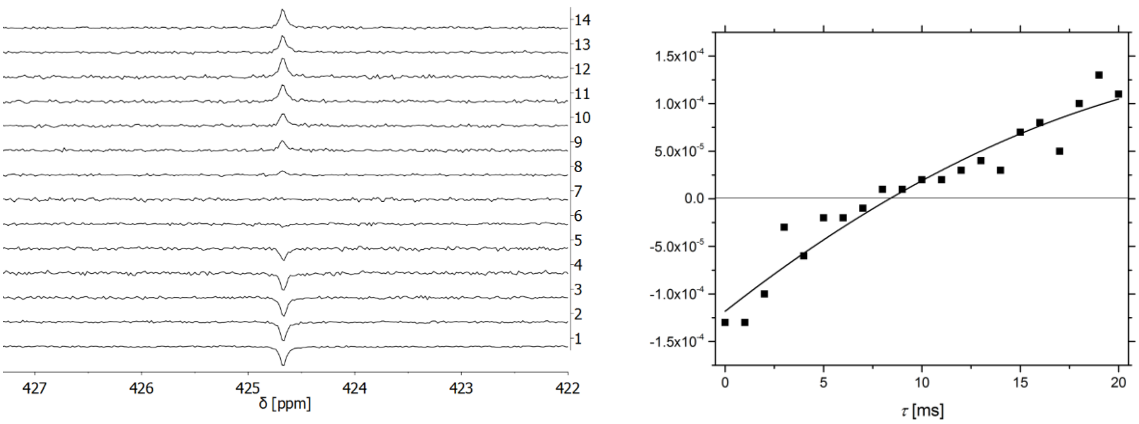

Figure S1.13. Left: Inversion recovery experiment from τ = 0 ms to τ = 14 ms of the ^{19}F NMR signal of elemental fluorine dissolved in $[(F_3C)_3CO]_2$ (**2b**) in a sealed PFA tube (external lock: [D6]Acetone, 376.13 MHz) at ambient temperatures. Right: Nonlinear fit [f(t) = f(inf) (1–H_0 exp(–t/T_1)), f(inf) =0.00016, T_1 = 13.5 ms, H_0 = 1.73045, χ^2 = 6.715507372E-9, σ = 0.00002 ms] of the intensities for the determination of T_1 of elemental fluorine dissolved in peroxide **2b.**

2. Computational Details

All structure optimizations and frequency calculations were carried out using restricted (closed shell species) Kohn-Sham density functional theory (RKS-DFT) with the B3LYP[13] hybrid exchange-correlation functional. To safe computational time, where applicable, the resolution-of-identity approximation RIJCOSX[14] was applied. The balanced basis sets of triple-ξ quality, TZVP, was used in these calculations.[15]

Table S2.1: Calculated reaction enthalpies $\Delta_R H$ of the decomposition of hypofluorites R^FOF at the B3LYP/aug-cc-pVTZ level of theory.

#						$\Delta_R H$ [kJ mol^{-1}]	ref.
a1	F_3COF	→	F_2	+	F_2CO	127.45	
a2	2 F_3COF	→	2 CF_4	+	O_2	–394.78	–332 [16]
b1	CF_3CF_2OF	→	F_2	+	$CF_3C(O)F$	127.43	
b2		→	CF_4	+	F_2CO	–390.81	
c	$CF_3CF_2CF_2OF$	→	C_2F_6	+	F_2CO	–397.19	–410.3 [17]
d1	$(F_3C)_2CFOF$	→	F_2	+	$(F_3C)_2CO$	150.85	
d2		→	CF_4	+	$CF_3C(O)F$	–392.91	
e	$(F_3C)_3COF$	→	CF_4	+	$(F_3C)_2CO$	–362.40	
f1	$(C_2F_5)(F_3C)_2COF$	→	CF_4	+	$(C_2F_5)(F_3C)CO$	–388.10	
f2		→	C_2F_6	+	$(F_3C)_2CO$	–387.73	

2.1 $(C_2F_5)(F_3C)_2COF$ – Rotational Isomers

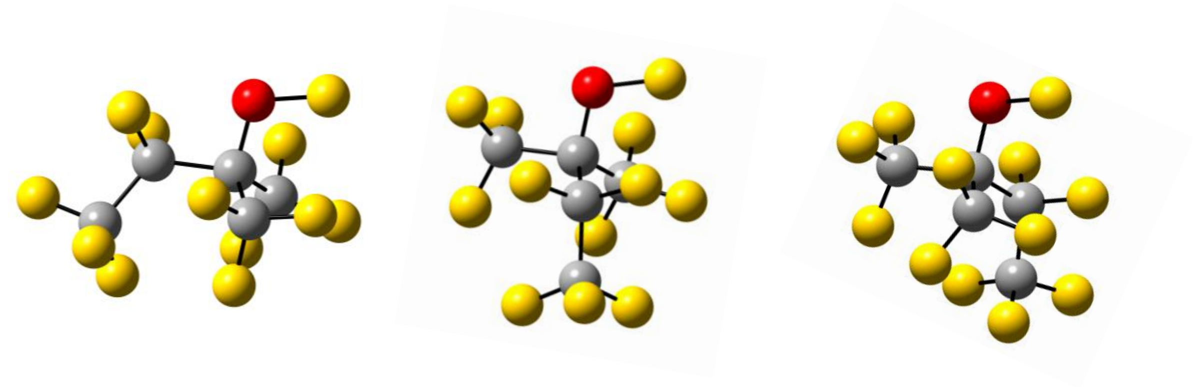

t-1 *t*-2 *t*-2‘

Figure S2.1. Computed minimum structures of the *trans* isomers of $(C_2F_5)(F_3C)_2COF$ (**1c**).

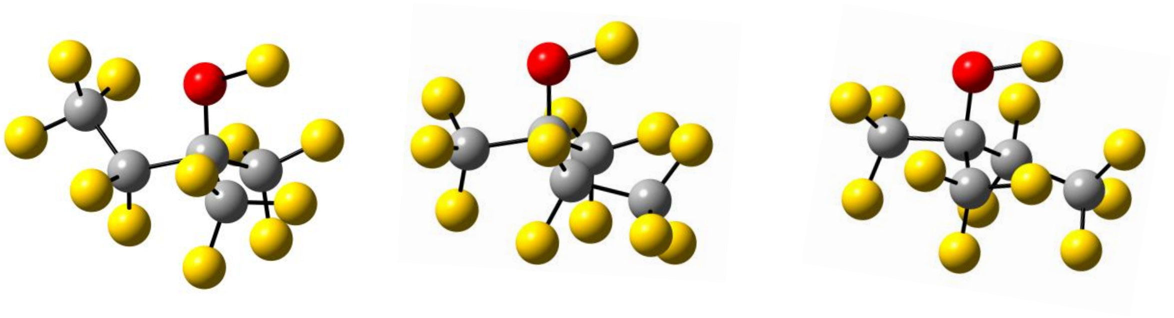

g-1 *g*-2 *g*-2’

Figure S2.2. Computed minimum structures of the *gauche* isomers of $(C_2F_5)(F_3C)_2COF$ (**1c**).

Table S2.2. Coordinates of the computed *trans* and *gauche* minimum structures of $(C_2F_5)(F_3C)_2COF$ (**1c**) obtained at the B3LYP/aug-cc-pVTZ level of theory.

isomer	atom	x	y	z	isomer	atom	x	y	z
t-1	C	-8.072137	-2.293379	2.602375	*g*-1	C	0.475794	2.330858	0.38541
	C	-9.295656	-1.538369	-0.011577		C	3.070879	2.099889	-1.128879
	F	-5.866984	-1.164391	3.013299		C	-1.742453	0.872038	-0.972588
	F	-7.692971	-4.78906	2.523696		C	-0.241231	5.216969	0.70302
	F	-9.627003	-1.787413	4.516799		O	1.08443	1.211214	2.723036
	F	-7.761736	-2.471318	-1.819409		F	-2.571514	2.178914	-2.972557
	C	-9.877926	1.343331	-0.717535		F	-0.981444	-1.40048	-1.764451
	C	-11.055963	2.87656	1.526425		F	4.485066	4.174593	-0.690604
	O	-11.728433	0.969892	-2.62385		F	2.540175	2.008223	-3.614511
	C	-7.465954	2.703305	-1.850875		C	4.870895	-0.192087	-0.497993
	F	-5.409187	2.184107	-0.479353		F	0.089527	6.455845	-1.476106
	F	-7.758138	5.209878	-1.86314		F	1.191141	6.282263	2.475174
	F	-7.066862	1.937725	-4.212802		F	-2.66053	5.475136	1.375937
	F	-12.952083	1.570964	2.564022		F	-0.873233	1.772949	4.501003
	F	-11.981301	5.088135	0.767062		F	-3.674578	0.521565	0.60562
	F	-9.293538	3.315045	3.29669		F	6.709905	-0.191647	-2.229343
	F	-12.088287	3.317517	-3.914886		F	3.693374	-2.411083	-0.578753
	F	-11.497953	-2.821336	-0.103291		F	5.912665	0.106281	1.77478
	E_{tot} = –1463.7444088240 H					E_{tot} = –1463.7475982050 H			
t-2	C	-8.820497	-1.475576	2.736298	*g*-2	C	1.655161	2.660922	0.808411
	C	-10.433647	-0.312279	0.523856		C	4.383882	2.186926	-0.253101
	F	-6.348101	-1.176708	2.37759		C	-0.396863	0.619181	-0.021823
	F	-9.322983	-3.949019	2.804051		C	0.694267	5.39198	-0.038232
	F	-9.449241	-0.474382	4.961904		O	1.720854	3.013973	3.448603
	F	-9.777581	-1.605817	-1.580468		F	-2.305744	0.739049	1.653049
	C	-10.34515	2.612318	-0.17027		C	0.287224	-2.265402	-0.293571
	C	-10.522958	4.422713	2.180206		F	-1.282487	1.294694	-2.328068
	O	-12.303734	3.104945	-1.932437		F	4.35421	1.948716	-2.767723
	C	-7.963855	3.33451	-1.845517		F	5.384077	0.090867	0.735169
	F	-5.834566	2.461492	-0.830505		F	5.889478	4.121651	0.349329
	F	-7.776693	5.840463	-2.052898		F	1.074518	5.759445	-2.497834
	F	-8.20162	2.378626	-4.16388		F	1.937848	7.178491	1.232054
	F	-12.231356	3.611001	3.843766		F	-1.761501	5.652762	0.455885
	F	-11.156332	6.749693	1.473308		F	-1.842197	-3.469516	-0.926973
	F	-8.269124	4.515365	3.341028		F	1.975976	-2.625198	-2.130915
	F	-14.63791	3.407693	-0.605821		F	1.158049	-3.2651	1.838348
	F	-12.853474	-0.889784	1.072774		F	2.256172	0.645672	4.631665
	E_{tot} = –1463.7449508120 H					E_{tot} = –1463.7456479760 H			
t-2’	C	1.485118	7.249442	0.180169	*g*-2’	C	1.252625	2.768701	0.690076
	C	2.758494	5.02853	-1.367402		C	3.787995	2.789381	-0.945362
	C	-1.504108	6.997776	0.379914		C	-0.602736	0.68258	-0.439637
	C	2.46762	9.874552	-0.870068		C	0.119963	5.507932	0.819225
	O	2.500249	6.76511	2.609057		O	2.256492	2.053194	3.052349
	F	-2.32502	8.781278	2.002642		F	-0.405477	0.758709	-2.992532
	C	-3.232205	7.214372	-2.026871		C	-3.531994	0.740392	0.088181
	F	-2.003763	4.718587	1.414296		F	0.219695	-1.580204	0.374813
	F	1.820258	4.900607	-3.700794		F	3.450044	4.011045	-3.118746
	F	2.376801	2.815761	-0.227751		F	4.534626	0.435572	-1.440206
	F	5.244999	5.413876	-1.527666		F	5.631453	3.938828	0.331725
	F	2.536813	9.851265	-3.39993		F	-0.736723	6.247136	-1.438736
	F	4.798333	10.310769	-0.026698		F	1.892577	7.137865	1.57595
	F	0.990347	11.783252	-0.150558		F	-1.787065	5.617689	2.469953
	F	-3.097474	5.143535	-3.450751		F	-4.520732	-1.295148	-1.036749
	F	-2.605757	9.22097	-3.424657		F	-4.591585	2.794509	-0.920311
	F	-5.617706	7.485593	-1.255087		F	-4.085123	0.640932	2.535094
	F	2.117769	8.944441	4.162822		F	0.218536	1.629824	4.773502
	E_{tot} = –1463.7449006530 H					E_{tot} = –1463.7456234640 H			

3. References

[1] R. K. Harris, E. D. Becker, S. M. Cabral de Menezes, P. Granger, R. E. Hoffman, K. W. Zilm, *Magn. Reson. Chem.* **2008**, *46*, 582-598.

[2] C. Cobas, S. Domínguez, N. Larin, I. Iglesias, C. Geada, F. Seoane, M. Sordo, P. Monje, S. Fraga, R. Cobas et al., *MestReNova*, Mestrelab Research S.L., **2015**.

[3] J. H. Prager, P. G. Thompson, *J. Am. Chem. Soc.* **1965**, *87*, 230-238.

[4] C. Lu, J.-H. Kim, D. D. Desmarteau, *J. Fluorine Chem.* **2010**, *131*, 17-20.

[5] J. H. Nissen, T. Stüker, T. Drews, S. Steinhauer, H. Beckers, S. Riedel, *Angew. Chem. Int. Ed.* **2019**, *58*, 3584-3588; *Angew. Chem.* **2019**, *131*, 3622-3626.

[6] *Recommendations on the transport of dangerous goods*. *Model regulations*, UNITED NATIONS, New York, **2007**.

[7] S. P. Kotun, J. D. O. Anderson, D. D. DesMarteau, *J. Org. Chem.* **1992**, *57*, 1124-1131.

[8] R. C. Kennedy, G. H. Cady, *J. Fluorine Chem.* **1973**, *3*, 41-54.

[9] I.-K. Suh, H. Ohta, Y. Waseda, *J Mater Sci* **1988**, *23*, 757-760.

[10] A. Williams, *J. Phys.: Condens. Matter* **1989**, *1*, 2569-2574.

[11] H. Ott, *Zeitschrift für Kristallographie - Crystalline Materials* **1926**, *63*.

[12] A. Jesih, K. Lutar, B. Žemva, B. Bachmann, St. Becker, B. G. Mller, R. Hoppe, *Z. Anorg. Allg. Chem.* **1990**, *588*, 77-83.

[13] a) A. D. Becke, *J. Chem. Phys.* **1993**, *98*, 5648-5652; b) C. Lee, W. Yang, R. G. Parr, *Phys. Rev. B* **1988**, *37*, 785-789; c) S. H. Vosko, L. Wilk, M. Nusair, *Can. J. Phys.* **1980**, *58*, 1200-1211; d) P. J. Stephens, F. J. Devlin, C. F. Chabalowski, M. J. Frisch, *J. Phys. Chem.* **1994**, *98*, 11623-11627.

[14] F. Neese, F. Wennmohs, A. Hansen, U. Becker, *Chem. Phys.* **2009**, *356*, 98-109.

[15] F. Weigend, R. Ahlrichs, *Phys. Chem. Chem. Phys.* **2005**, *7*, 3297-3305.

[16] V. Francesco, M. Sansotera, W. Navarrini, *J. Fluorine Chem.* **2013**, *155*, 2-20.

[17] W. Navarrini, V. Tortelli, A. Russo, S. Corti, *J. Fluorine Chem.* **1999**, *95*, 27-39.
